# Intra-articular injections of platelet-rich plasma in symptomatic knee osteoarthritis: a consensus statement from French-speaking experts

**DOI:** 10.1007/s00167-020-06102-5

**Published:** 2020-06-24

**Authors:** Florent Eymard, Paul Ornetti, Jérémy Maillet, Éric Noel, Philippe Adam, Virginie Legré-Boyer, Thierry Boyer, Fadoua Allali, Vincent Gremeaux, Jean-François Kaux, Karine Louati, Martin Lamontagne, Fabrice Michel, Pascal Richette, Hervé Bard, the GRIP (Groupe de Recherche sur les Injections de PRP, PRP Injection Research Group)

**Affiliations:** 1 Department of Rheumatology, AP-HP Henri Mondor Hospital, 94010 Créteil Cedex, France; 2 Department of Rheumatology, Plateforme d’Investigations Technologiques Dijon University Hospital, INSERM 1093 CAPS, Dijon, France; 3 Department of Rheumatology, AP-HP Lariboisière Hospital, 75010 Paris, France; 4 Santy Orthopedic Center, 69008 Lyon, France; 5 Imaging Department, Medipole Garonne Sport Clinic, 31100 Toulouse, France; 6 American Hospital Paris, 92200 Neuilly-sur-Seine, France; 7 IAL Nollet, 75017 Paris, France; 8 Department of Rheumatology, El Ayachi Hospital, Salé, Morocco; 9 Sport Medicine Unit, Division of Physical Medicine and Rehabilitation, Swiss Olympic Medical Center, Lausanne University Hospital, Lausanne, Switzerland; 10 Physical, Rehabilitation Medicine and Sports Traumatology, SportS2, FIFA Medical Centre of Excellence, IOC Research for Prevention of Injury and Protection of Athlete Health, FIMS Clinical Centre of Sports Medicine, University and University Hospital of Liège, 4000 Liège, Belgium; 11 Department of Rheumatology, AP-HP Saint-Antoine Hospital, 75012 Paris, France; 12 Montreal University Hospital Center, Montreal, Canada; 13 Physical Medicine and Rehabilitation Department, CHRU hôpital Jean-Minjoz, 25000 Besançon, France; 14 Department of Rheumatology, AP-HP Lariboisière Hospital, 75010 Paris, France; 15 Cabinet médical Vaudoyer, 75007 Paris, France

**Keywords:** Knee osteoarthritis, Platelet-rich plasma, Clinical practice, Consensus, Intra-articular injections

## Abstract

**Purpose:**

There has been much debate regarding the use of intra-articular injections of platelet-rich plasma (PRP) as symptomatic treatment for knee osteoarthritis. The objective of this consensus was to develop guidelines for PRP injections in knee osteoarthritis according to the French National Authority for Health recommendations.

**Methods:**

Fifteen physicians from different French-speaking countries (10 rheumatologists, 4 specialists in rehabilitation and sports medicine and 1 radiologist) were selected for their expertise in the areas of PRP and osteoarthritis. A comprehensive literature review was conducted on Medline including all published therapeutic trials, open studies, meta-analysis and systematic reviews focusing on the effects of PRP in knee OA, as well as fundamental studies concerning the characteristics of the various types of PRP and their mechanisms, indexed before April 2019. Using the method recommended by the French National Authority for Health inspired by the Delphi consensus process, 25 recommendations were finally retained and evaluated. The recommendations were classified as appropriate or not appropriate, with strong or relative agreement, or uncertain if a consensus was not achieved.

**Results:**

Among the 25 recommendations selected, the main ones are the following: (1) Intra-articular injections of PRP are an effective symptomatic treatment for early to moderate knee osteoarthritis. This recommendation was considered appropriate with a relative agreement (Median = 8; rank = 6–9). Level of evidence 1A. (2) A PRP treatment sequence in knee osteoarthritis may include 1–3 injections. This recommendation was considered appropriate with a strong agreement (Median = 9; rank = 7–9). Level of evidence 1A. (3) Leucocytes-poor PRP should be preferred in knee osteoarthritis. This recommendation was considered appropriate with a relative agreement (Median = 8; rank = 5–9). Level of evidence 5. (4) Intra-articular PRP knee injections should be performed under ultrasound or fluoroscopic guidance. This recommendation was considered uncertain with no consensus (Median = 8; rank = 3–9). Level of evidence 5. (5) PRP should not be mixed with an anesthetic or intra-articular corticosteroid. This recommendation was considered appropriate with a relative agreement (Median = 9; rank = 6–9). Level of evidence 5

**Conclusion:**

Those 25 recommendations should standardize and facilitate the use of IA PRP injections, which are considered by experts as an effective treatment especially in early or moderate knee OA. Although a strong or relative agreement from the experts was obtained for most of the recommendations, many of them had a very low level of evidence (Level 5) and were principally based on the clinical experience of the experts.

**Electronic supplementary material:**

The online version of this article (10.1007/s00167-020-06102-5) contains supplementary material, which is available to authorized users.

## Introduction

In recent years, there has been a significant increase in the use of platelet-rich plasma (PRP) injections for the treatment of several musculoskeletal diseases, especially in knee osteoarthritis (OA). PRP is obtained from an autologous blood sample and platelets are concentrated either by apheresis or by centrifugation [24]. The activation of platelets contained in the PRP induces the release of a large number of mediators by the platelet granules which may contain up to 800 protein components (granule secretion) [96]. Among the several components released are many growth factors [Transforming Growth Factor Beta (TGFβ), Hepatocyte Growth Factor (HGF), Vascular Endothelial Growth Factor (VEGF), Platelet Derived Growth Factor (PDGF), Insulin Growth Factor (IGF), Fibroblast Growth Factor (FGF), Connective Tissue Growth Factor (CTGF)] but also soluble anti-inflammatory mediators [(interleukin receptor antagonist (IL)-1 (IL1-RA), IL-4, IL-8, IL-8, IL-10, etc.)] that may contribute to the therapeutic effects. Preclinical studies have shown that the granule secretions have mainly a homeostatic effect with anabolic and anti-inflammatory impacts on joint tissues and cells [16, 30, 50, 57, 64, 71, 92].

Recently, international scientific societies such as American College of Rheumatology (ACR) [52] and Osteoarthritis Research Society International (OARSI) [5] have recommended not using PRP in symptomatic knee OA. They emphasized about the absence of enough robust data concerning clinical efficiency and the heterogeneity of PRP preparations. Even if the quality of clinical studies assessing PRP in knee OA could be discussed, results of several therapeutic trials and meta-analysis already published should be considered. The majority of published studies compared PRP with placebo (saline, NaCl) or hyaluronic acid (HA). Placebo-controlled trials [37, 56, 74, 90] found that the efficacy of PRP was significantly better after a follow-up between 3 and 12 months, while randomized trials comparing PRP to HA [3, 8, 13, 17, 23, 26, 33, 37, 43, 54, 56, 58, 78, 77, 88, 91, 94] suggested that PRP would have at least equivalent or greater efficacy than HA in symptomatic knee OA. On the other hand, the heterogeneity of protocols from one study to another limits the extrapolation of their results. This heterogeneity is related to the different methods of PRP preparation (number of centrifugation, type of kit used to separate the platelet concentrate, leukocyte or red cell concentration, etc.), to the number of injections and the volume injected, and also to the specific recommendations after injections (duration of relative rest and NSAID interruption, for example) [3, 8, 23, 33, 34, 37, 54, 58, 74, 91]. The creation of a group of experts with clinical experience and scientific expertise of PRP, a thorough knowledge of knee OA and involvement in clinical and/or fundamental research was warranted as such a group could combine the heterogeneous data from the literature and their experience as practitioners. For that purpose that a group of French-speaking experts (Groupe de Recherche sur les Injections de PRP; PRP Injection Research Group) was set up in 2018, with the specific goal to develop the first international consensus for the use of PRP in knee OA according to the current recommendations from the French National Authority for Health in order to homogenize clinical practices, help practitioners and avoid misuse.

## Materials and methods

### Experts

Fifteen French-speaking experts from 5 countries (Belgium, Canada, France, Morocco and Switzerland) were invited to participate. This group included 10 rheumatologists (FE, PO, JM, EN, VLB, TB, FA, KL, PR, HB), 4 specialists in rehabilitation and sports medicine (VGB, JFK, ML, FM) and 1 radiologist (PA) working in public or private medical centers. The experts were selected given to their scientific expertise and clinical experience of PRP injections in musculoskeletal diseases and especially in knee OA, their thorough knowledge of literature data and their involvement in clinical and/or fundamental research in OA and/or PRP.

### Methodology of the recommendations

The methodology used to draft this consensus was based on the current recommendations of the French National Authority for Health, which are inspired by the Delphi method [39]. A first working group (FE, PO, JM, EN, PA, VLB, TB, HB) was formed to conduct a comprehensive literature review of all published therapeutic trials, open studies, meta-analysis and systematic reviews focusing on the effects of PRP in knee OA (injection protocols, analgesic effect, structural effect and side effects), as well as fundamental studies concerning the characteristics of the various types of PRP and their mechanisms. The search was conducted on Medline and included articles of interest indexed before April 2019. The main elements of the literature review were presented to the whole group at 2 different meetings. This resulted in the development of 43 proposed recommendations written in French. Recommendations were grouped into 7 different themes (indications, choice of PRP, therapeutic protocol, contraindications, post-injection management, tolerance, miscellaneous). At the end of the second meeting, the number of recommendations were reduced to 29. Indeed, fourteen were withdrawn because they were considered redundant or less relevant (Supplementary Table 1).

A second group of experts (FA, VGB, JFK, KL, ML, FM, PR) have been added and all 15 experts voted anonymously for each of the 29 recommendations. During a third working meeting, experts reviewed all recommendations in order to compare their points of view both on the interest and on the wording of each of them. Following this meeting, 4 additional recommendations were excluded because deemed redundant by the experts or because, neither the literature nor the expert practice made it possible to provide any elements of response (Supplementary Table 2) and 16 were reformulated to avoid any risk of confusion in their wording. At the end, 25 recommendations were retained for a second vote (Supplementary Fig. 1). The results are presented in this article.

### Rating of recommendations

The rating of each recommendation was done according to the guidelines from the French National Authority for Health [39]. Each expert rated each recommendation between 1 and 9 (from “totally inappropriate” to “totally appropriate”); a value of “5” indicated uncertainty. The scores of all the experts were pooled, and the median was calculated. A recommendation was considered appropriate when the value of the median was ≥ 7, either with a strong agreement if the distribution of ratings was in the (7–9) range or with a relative agreement if the distribution of ratings was in the (5–9) range. A recommendation was considered inappropriate when the value of the median was ≤ 3.5, associated with strong agreement [distribution of ratings in the (1–3) range] or relative agreement [distribution of ratings in the (1–5) range]. The recommendation was considered uncertain when the value of the median was between 4 and 6.5 (indecision) and a lack of consensus was stated in any other situation not described above. The level of evidence depending of study design was notified for each recommendation (from 1A, best evidence to 4 5, weak evidence) [10]: 1A, Systematic review (with homogeneity) of RCTs; 1B, Individual RCT (with narrow confidence intervals); 2A, Systematic review (with homogeneity) of cohort studies; 2B, Individual Cohort study (including low quality RCT, e.g. < 80% follow-up); 3A, Systematic review (with homogeneity) of case–control studies; 3B, Individual Case–control study; 4, Case series (and poor quality cohort and case–control study); 5, Expert opinion without explicit critical appraisal or based on physiology bench research or “first principles”.

## Results

The recommendations are detailed below and summarized in Tables 1, 2, 3, 4, 5. For each recommendation, the median, the distribution of the expert scores, the appropriateness, inappropriateness or uncertainty of the recommendation, and the strong or relative agreement among the experts are specified.

1. Intra-articular injections of PRP are an effective symptomatic treatment for early to moderate knee osteoarthritis.
  Most clinical trials assessing the efficacy of PRP injections included subjects with radiographically mild to moderate knee OA [Kellgren-Lawrence (KL) grade II and III] [49] whereas patients with severe OA (KL grade IV or Ahlbäck stage IV/V [2]) were most often excluded. Despite some methodological limitations, all randomized, placebo-controlled studies demonstrated that the efficacy of PRP was statistically superior to NaCl on pain and function and clinically relevant up to 6 to 12 months after injections according to several outcome measures [VAS, International Knee Documentation Committee (IKDC), Western Ontario and McMaster Universities Osteoarthritis Index (WOMAC)] [37, 56, 74, 90]. Many trials have also compared the effect of PRP and HA injections on similar populations, showing either similar results or superiority of PRP compared to HA [17, 23, 33, 37, 43, 54, 58, 87, 94]. Conversely, none of these studies showed a superiority of HA. Several meta-analyses confirmed that PRP was superior to NaCl and at least as effective as HA, and possibly even superior in the long term (12 months) [14, 21, 41, 47, 55, 85, 89, 98, 102]. According to these data, experts agree that PRP injections are an effective treatment for mild to moderate knee OA. On the other hand, radiographic severity appeared to be a factor in poor response to PRP [14].
  Level of evidence 1A (Table 1).
  Median = 8; rank = 6–9.
  Expert opinion: appropriate with relative agreement.
2. Intra-articular injections of PRP may be useful in severe knee osteoarthritis (Kellgren and Lawrence grade IV).
  Even if few studies have focused specifically on severe knee OA, most available literature data suggest a negative impact of radiographic severity on the efficacy of PRP. In a post hoc analysis of a randomized controlled trial, the efficacy of PRP in subjects with grade IV OA was reduced as compared to subjects with moderate OA (KL ≤ III) but remained significantly better than NaCl at 6 months [37]. Similarly, a trial comparing 3 PRP injections and 3 low or high molecular weight HA injections showed a blunting effect of the 3 types of injections in patients with the most severe forms of OA [54]. Another study comparing 2 types of PRP, with different platelet and leukocyte concentrations, also found that both products were less effective in more severe forms of OA [31]. Only one small randomized controlled trial specifically compared a single injection of Leucocyte-Poor PRP (LP-PRP) and a CS injection in advanced OA (grade III or IV). Nearly 75% of patients reported a very significant or significant improvement after PRP injections with a mean decrease in VAS pain at 3 months of 41 out of 100 points without statistically difference with CS [46]. These data are in favor of a potential symptomatic effect in patients with severe OA. Therefore, experts have considered that PRP injections could be discussed in patients with severe OA, especially for patients who cannot undergo surgery due to comorbidities or because they do not want arthroplasty. However, clinicians should not expect the same clinical benefit after PRP injections in subjects with severe OA as compared to patients with early OA.
  Level of evidence 2B (Table 1).
  Median = 7; rank = 6–7.
  Expert opinion: appropriate with relative agreement.
3. Age, weight and physical activity can influence the indication and outcome of intra-articular injections of PRP in knee osteoarthritis.
  Few studies have examined predictive factors for a response to PRP injections. Age is the main factor studied and being older appears to be a factor of poor response [31, 44, 54, 74]. Several in vitro studies have shown a negative correlation between age and the amount of growth factors contained in the PRP [27, 72, 93, 97] and O’Donnel et al*.* showed that the stimulation of chondrocytes by PRP from elderly OA patients induced a catabolic cellular phenotype [72]. A few uncontrolled open-label studies have suggested that overweight has a negative impact on the symptomatic efficacy of PRP injections [32, 53] as already shown with HA [28], while a randomized placebo-controlled trial did not find a correlation between BMI and clinical response [74]. Even if there is no high-quality evidence regarding the impact of physical activity on the effectiveness of the PRP, a previous study showed that HA injections were more effective for pain when combined with a physical activity program [84]. Considering these data, the experts agreed that certain clinical parameters and physical activity levels could have an impact on the effectiveness of PRP treatment.
  Level of evidence 4 (Table 1).
  Median = 8; rank = 5–9.
  Expert opinion: appropriate with relative agreement.
4. The topographic pattern of the osteoarthritis influences the outcome of PRP treatment in knee osteoarthritis.
  To our knowledge, no studies have compared the effect of PRP according to the OA location in femorotibial joint (lateral vs. medial compartment). On the other hand, Jang et al*.* showed that the existence of patellofemoral OA was associated with a poorer clinical response [44], as already observed for HA injections [18].
  No consensus could be reached in the expert group due to a lack of sufficient data in the literature. Further studies evaluating topographic pattern of the knee OA as predictive factors for good or poor response to PRP injections are expected.
  Level of evidence 4 (Table 1).
  Median = 7; rank = 4–9.
  Expert opinion: uncertain. Lack of consensus.
5. PRP treatment should be offered as a second line of treatment after failure of oral or non-pharmacological treatment for knee osteoarthritis.
  The experts consider that, like other injectable symptomatic treatments, PRP should be offered as a 2nd line of treatment after failure of a pharmacological treatment including conventional analgesics and/or NSAIDs and non-pharmacological management based on appropriate physical activity and/or physiotherapy and lifestyle changes. Beyond this general principle, the management of knee OA must be personalized, particularly because the associated comorbidities are often numerous. In this context, autologous PRP injections have a better safety profile than the majority of oral pharmacological treatments [19, 20, 83] (see recommendations 23 and 24).
  Level of evidence 5 (Table 2).
  Median = 9; rank = 5–9.
  Expert opinion: appropriate with relative agreement.
6. PRP treatment should not be used during a flare-up of knee osteoarthritis.
  Experts consider that treatment with PRP injection is not an appropriate treatment during an acute inflammatory phase of knee OA [65], unlike injectable CS. Nevertheless, it is important to note that some studies have shown that PRP reduced effusion [15], the extent of synovitis [79] and decreased the level of catabolic and inflammatory mediators in synovial fluid [15, 101].
  Level of evidence 5 (Table 2).
  Median = 7; rank = 5–9.
  Expert opinion: appropriate with relative agreement.
7. The characteristics of the injected PRP influence the result in knee osteoarthritis.
  There is not one but several types of PRP, whose characteristics (number of platelets and leukocytes and the amount of growth factors, pro- or anti-inflammatory cytokines) vary with the protocol used to obtain it but also depend to specificities of each patient and can vary over time [66]. Only the apheresis technique can obtain PRPs with identical platelets and white blood cell concentrations in all patients.
  Milants et al*.* compared the characteristics of PRP used in randomized controlled trials that showed very good symptomatic response [2 × MCII (minimal clinically important improvement)] and those used in trials that found a modest effect (< 1 × MCII) [69]. They analyzed the protocols of preparation (type of centrifugation) and injection (volume and number of injections), but also the PRP composition (leukocyte and platelet concentrations). The use of double centrifugation, a platelet concentration greater than 5 × normal and a high leukocyte count were predictive of poor response. Moreover, a recent in vitro study showed that PRP from elderly subjects with knee OA induced an inflammatory phenotype on cultured macrophages and a catabolic phenotype on chondrocytes in three-dimensional culture, unlike PRP in young subjects without OA [72]. These data therefore support the impact of the composition of PRP on the clinical response obtained after injection. Specific impact of PRP composition and injection protocols are discussed below (recommendations 8, 11, 12, 13).
  Level of evidence 4 (Table 3).
  Median = 8; rank = 6–9.
  Expert opinion: appropriate with relative agreement.
8. Leucocyte-poor PRP should be preferred in knee osteoarthritis.
  The complexity of the composition of PRP but also of the specific effect of each component on homeostasis of joint tissues makes it extremely difficult to draw a clear and definitive conclusion on the specific effect of a particular component of the PRP such as leukocytes. This is all the more complex since the joint tissue will certainly not respond in the same way depending on the severity of OA process. Leukocytes, whose number greatly depends on the protocol, could modulate the effect of PRP by promoting a local inflammatory reaction, which could have a beneficial effect on the wound process particularly sought in chronic tendinopathy but could also increase the painful symptoms. To our knowledge, only one study has compared injections of leukocyte-rich PRP (LR-PRP) (× 1.4 blood concentration) and PRGF (Plasma Rich Growth Factors) that did not contain leukocytes in knee OA. No difference was found in terms of pain and functional symptoms [31], but there was more post-injection pain and swelling in the LR-PRP group. Riboh et al*.* conducted a network meta-analysis to indirectly compare low LP-PRP and LR-PRP. They found no difference in efficacy or adverse events between the two types of PRP, but only LP-PRP was statistically superior to placebo and HA [81]. A rabbit study showed that intra-articular injection of LR-PRP induced an inflammatory profile of the synovial fluid contrary to LP-PRP, which appeared to be dependent on the NFkB pathway [99]. In addition, the structural benefit observed after LP-PRP was significantly greater than after injection of LR-PRP. On the other hand, a similar study led in humans did not demonstrate any induction of an inflammatory profile (IL-6, IL-1β, IL-17A and IL-8) in plasma and synovial fluid after LR-PRP injections [63]. In total, considering the higher level of evidence regarding the efficacy of LP-PRP in knee OA, the persistence of a gray area on the over-risk of adverse events, particularly post-injection inflammatory reactions, with LR-PRP and some results of preclinical data, experts consider that it is justified preferring LP-PRP for knee OA while recognizing the weakness of the available evidences.
  Level of evidence 5 (Table 3).
  Median = 8; rank = 5–9.
  Expert opinion: appropriate with relative agreement.
9. A blood count should be obtained less than 3 months before PRP treatment.
  The advantage of obtaining a recent full blood count lies as much in the potentially predictive role of the number of circulating platelets as in the identification of potential contraindications. Indeed, several studies have shown a link between the number of platelets and growth factors contained in the PRP [58, 76, 93]. However, the relationship between platelet quantity and clinical response is more uncertain and non-linear [22, 58]. Even if no study has demonstrated that injecting PRP from subjects with a disease affecting the platelet line or another cell line was ineffective or dangerous, it seems reasonable to take a precautionary approach. According to the experts, a recent full blood count (less than 3 months old) is indicated before a series of PRP injections.
  Level of evidence 5 (Table 5).
  Median = 8; rank = 6–9.
  Expert opinion: appropriate with relative agreement.
10. PRP injections should follow the same traceability rules as other injectable therapeutic devices.
  Lacking specific literature, the experts agree on the need for traceability of PRP kits as for other injectable devices. As the main concern is the occurrence of infection, it is important that the batch can be traced for kit used. Similarly, batch identification is required if one of the kit’s components (tube, syringe, separator gel, sampling equipment) is found to be defective.
  Level of evidence 5 (Table 5).
  Median = 9; rank = 7–9.
  Expert opinion: appropriate with strong agreement.
11. A PRP treatment sequence in knee osteoarthritis may include 1–3 injections.
  In the literature, there is no consensus regarding the number of injections to be performed during a therapeutic “cycle”. Based on HA injection protocols, the majority of trials published to date included 3 injections per week [33, 37, 56, 63, 88, 90, 94] or every 2 weeks [3, 54, 70]. Nevertheless, several trials have demonstrated the efficacy of a single PRP injection as compared to injections of NaCl [74], HA [8, 37, 58] or CS [46] in knee OA. Görmeli et al*.* showed that 3 weekly injections of LP-PRP were more effective than a single injection in early knee OA [37]. Another study comparing 1, 2 and 3 LR-PRP injections every 2 weeks showed that patients who received at least 2 injections reported superior efficacy as early as the first month [48]. Finally, another trial reported that the “cycle” of 3 monthly injections of LP-PRP had superior efficacy than 1 or 2 injections over 12 months [42]. Conversely, Patel et al*.* found no difference between 1 and 2 LP-PRP injections at 3-week intervals over a 6-month follow-up period [74]. A recent meta-analysis showed that multiple PRP injections (especially 3) in knee OA were more effective than a single injection to improve joint function but not pain [95]. The absence of statistically significant effect of multiple injections on pain (Standard Mean Difference = 0.65 (− 0.31, 1.60); *p* = 0.19) was especially related to the negative results of Patel et al. Currently, several authors recommend at least 2 subsequent injections of PRP [68]. However, also taking into account the cost of treatment for the patient and the current low level of evidence, the expert group did not establish a minimum number of PRP injections to be made, but consider that a “cycle” of PRP may include 1 to 3 injections spaced by 1 to 4 weeks apart.
  Level of evidence 1A (Table 2).
  Median = 9; rank = 7–9.
  Expert opinion: appropriate with strong agreement.
12. The volume of a PRP injection in knee osteoarthritis should be 4–8 mL.
  The effectiveness of IA PRP injections can potentially be modulated by the absolute value of injected platelets and by extension the rate of growth factors and cytokines contained in the PRP which depend on the volume of PRP injected [61]. In randomized trials, the average volume injected was 5 mL [35]. An uncontrolled open-label study showed the efficacy of a single injection of PRP with an average volume of 8.8 mL [40]. The choice of this volume was justified by the distribution volume of the knee joint cavity which was recently estimated at 9 mL [80]. Experts believe that the injection of a volume of PRP of 4 to 8 mL is appropriate, but it ultimately remains dependent on the kit used for its extraction.
  Level of evidence 5 (Table 3).
  Median = 8; range = 7–9.
  Expert opinion: appropriate with strong agreement.
13. The efficacy of PRP in knee osteoarthritis depends on the number of platelets injected.
  Even if the effect of PRP remains incompletely elucidated, certainly complex and multifactorial, it is currently accepted that the numerous growth factors such as PDGF, TGF-β, VEGF, IGF, FGF but also pro- and anti-inflammatory cytokines released after activation of platelets play a central role related to homeostatic effect on joint tissues [73]. Indeed, several studies showed an anabolic effect of PRP on cartilage tissue [16, 30] and chondrocyte metabolism [30, 57, 71]. The role of PRP on synoviocyte metabolism appeared more complex with both pro- and anti-inflammatory effects depending in part of PRP characteristics [4, 7, 64, 92] although in vivo studies brought out rather an anti-inflammatory effect of PRP on synovial tissue [16, 50]. Some preliminary studies in humans also seem to show beneficial homeostatic effects of PRP injections on joint tissues [3, 29, 79]. The correlation between the number of platelets and the quantity of growth factors released in the injected PRP has been clearly demonstrated [58, 60, 76, 93, 99]. In contrast, the correlation between the number of platelets in the PRP and clinical response is not clear [22, 31, 58]. In a study recently published by Louis et al*.* the platelet count was positively correlate with PDGF and TGF-β levels but did not influence the clinical efficacy [58]. According to a recent meta-analysis, excessive platelet concentration (> 5 N) may decrease the efficacy of PRP [21]. These results illustrate the complexity of PRP mechanism of action in OA However, experts agree that the composition of PRP plays a central role in its therapeutic effectiveness, and that platelets play the most active role. Yet, a number of unknowns prevent an optimal platelet threshold from being defined.
  Level of evidence 4 (Table 3).
  Median = 8; rank = 5–9.
  Expert opinion: appropriate with relative agreement.
14. PRP should not be mixed with an anesthetic or intra-articular corticosteroid.
  Several studies have shown that PRP induced significant cell proliferation in tenocyte or chondrocyte cell culture models [12, 25, 45]. Inversely, CS and local anesthetics inhibited proliferation, induced excess cell death but also partially or totally inhibited the beneficial effect of PRP. The toxic effect of local anesthetics on platelet function, particularly platelet aggregation, is also well demonstrated [36, 38, 75]. By extension, we can suspect that anesthetics have a negative effect on platelet activation and the release of growth factors and cytokines. Experts agree that the concomitant use of local anesthetics, particularly in IA injection, should be avoided, as should the simultaneous injection of CS.
  Level of evidence 5 (Table 4).
  Median = 9; rank = 6–9.
  Expert opinion: appropriate with relative agreement.
15. Anti-inflammatory treatment should be avoided in the days before and after PRP treatment.
  NSAIDs inhibit platelet aggregation by inhibiting cyclooxygenases. This inhibition is reversible for all NSAIDs except aspirin and its length is dependent on the half-life of each NSAID. Recently, an animal study showed that the addition of a selective COX2 inhibitor did not alter the release of growth factors (TFG β, PDGF BB) or expression of platelet activation markers (CD62P, CAP1) [59]. In contrast, a study in healthy subjects showed that daily use of naproxen significantly decreased the amount of certain growth factors such as PDGF AA and AB until one week after naproxen was discontinued [62]. There is a lack of comparative clinical data to assess the impact of concomitant NSAID use during PRP therapy. Inversely, the interruption of NSAIDs can lead to an increase in pain. Therefore, NSAIDs should be interrupted for the shortest period possible. Based on the available data, our group recommends an interruption of NSAIDs before and after PRP injection but does not provide a specific duration. A one-week break before and after injection appears reasonable. The literature does not provide sufficient evidence to recommend the use of one NSAID over another, however, the safety of the continuation of a COX2 inhibitor identified by Ludwig et al. merits further investigation [59].
  Level of evidence 5 (Table 4).
  Median = 9; rank = 7–9.
  Expert opinion: appropriate with strong agreement.
16. Treatment of knee osteoarthritis with PRP should be done away from an intra-articular injection of a corticosteroid.
  The association of a CS with PRP has a negative impact on the anabolic properties of PRP in vitro [12, 25, 45]. Therefore, it is not recommended to inject CS and PRP simultaneously. On the other hand, a two-step therapeutic protocol involving first an injection of CS and then PRP may be considered, particularly in the case of joint effusion and a fortiori of acute inflammation phase in knee OA. To our knowledge, only one study comparing a single injection of PRP with sequential treatment by methylprednisolone injection followed by PRP one week later showed that this protocol yielded a better clinical response during the first 3 months than PRP alone [11]. If CS infiltration is considered after injection of PRP, it seems reasonable to wait a minimum of one week given the estimated platelet life of 7 to 10 days. The experts also recommend a delay when a CS is injected before PRP without specifying the duration given the limited data in the literature.
  Level of evidence 5 (Table 4).
  Median = 8; rank = 5–9.
  Expert opinion: appropriate with relative agreement.
17. Intra-articular PRP knee injections should be performed under ultrasound or fluoroscopic guidance.
  Only about 80% of injections performed under anatomical landmarks alone were successfully delivered in the IA space [6, 9]. In a recent comparative study, the rate of IA injection on an OA knee without effusion increased from 83.7% without guidance to 96% under ultrasound [9]. On the other hand, ultrasound does not pose any risk to the patient, unlike fluoroscopic examination, which uses radiation. While the median of the expert votes was clearly in favor of the use of radiological guidance, there was no consensus on this recommendation due to the low score of one expert (score = 3) who considered that IA injections into the knee could continue to be delivered using only anatomical landmarks.
  Level of evidence 5 (Table 2).
  Median = 8; rank = 3–9.
  Expert opinion: uncertain. Lack of consensus.
18. Joint effusion should be systematically drained before PRP injection.
  Despite the absence of specific data in the literature, there is a consensus among experts on this recommendation. The interest of draining effusion before an injection is undeniable, allowing an immediate improvement in pain and functional limitations. It also prevents dilution of the PRP at the time of injection.
  Level of evidence 5 (Table 2).
  Median = 9; rank = 7–9.
  Expert opinion: appropriate with strong agreement.
19. Recent neoplasia (malignant tumors, hematological diseases) may be a contraindication to intra-articular PRP injections.
  There is no demonstrated link in the literature between the contents of PRP and the risk of tumor proliferation, either locally or remotely. The theoretical risk of promoting tumor growth by injecting PRP, via the addition of growth factors directly into a joint that is the site of a benign (villonodular synovitis, primary osteochondromatosis) or malignant tumor (sarcoma) contraindicates treatment with PRP. Pending further data, this recommendation also applies to solid tumors with or without metastasis located at a distance from the knee. The experts also advised against the use of IA injections of PRP in patients with hematological diseases, particularly those that affect platelet production as they could modify the biological and cellular properties of PRP and thus alter its efficacy (see recommendation 9).
  Level of evidence 5 (Table 4).
  Median = 7; rank = 5–9.
  Expert opinion: appropriate with relative agreement.
20. Antiplatelet aggregation therapy is not a contraindication to PRP injections but may alter its efficacy by preventing platelet activation.
  Antiplatelet drugs, mainly aspirin and clopidogrel, are irreversible inhibitors of platelet aggregation. They can therefore limit platelet activation and thus limit the action of PRP. However, no study has investigated their actual impact on the effect of intra-articular PRP injections. Considering the major importance of these therapies in patients with cardiovascular diseases and the risk of severe complications during interruption of treatment, even transient, the experts have considered that it was preferable not to interrupt antiplatelet drugs before PRP injections. In some cases of primary prevention and after discussion with the cardiologist, a transient interruption of antiplatelet therapy could be considered. The duration of interruption is not codified but could, as an indication, be one week before up to one week after PRP injection taking into account the elimination half-life of the drugs as well as the platelet lifetime.
  Level of evidence 5 (Table 4).
  Median = 9; rank = 7–9.
  Expert opinion: appropriate with strong agreement.
21. The presence of radiographic chondrocalcinosis is not a contraindication to intra-articular injections of PRP.
  The prevalence of chondrocalcinosis in the knee is estimated to be 7% in adults and steadily increases with age [1]. Episodes of acute crystal-induced arthritis have been reported after several types of injections, and particularly after injections of HA. No association, other than fortuitous, was found between the injection procedure and the inflammatory episode [82]. The expert group therefore considers that radiographic chondrocalcinosis is not a contraindication. On the other hand, the patient should be informed of the very low risk of acute crystal-induced arthritis following the injection.
  Level of evidence 5 (Table 4).
  Median = 8; range = 7–9.
  Expert opinion: appropriate with strong agreement.
22. After injection of PRP, it is recommended to rest the knee for 48 h.
  With the exception of one vote, the experts recommended 48 h of rest following the injection of PRP. Of course, this is not strict bed rest, but a restriction of physical activities for 48 h. Patients should avoid any sports activity, prolonged walks, or carrying heavy loads. The duration of 48 h was based on pragmatism and clinical experience of experts. No literature data was available on this topic. Given the disagreement of one of the members, this recommendation was classified as uncertain.
  Level of evidence 5 (Table 2).
  Median = 9; rank = 3–9.
  Expert opinion: uncertain. Lack of consensus.
23. Intra-articular PRP injections in knee osteoarthritis are a locally well-tolerated treatment.
  All controlled trials, open trials and meta-analyses provided very reassuring data on the local tolerance of PRP injections. Post-injection local pain and/or swelling occurred in less than 10% of cases [14, 51]. The vast majority of meta-analysis did not find a statistically significant increase in adverse events after injection of PRP compared with other injected products [14, 21, 41, 47, 55, 89]. Only the meta-analysis published by Khoshbin et al*.* found an overall increase in adverse events after PRP vs. controls (8.4% vs. 3.8%) [51]. To date, no cases of septic arthritis have been reported after IA injection of PRP. Some studies suggest that the most platelet-concentrated and/or LR-PRP would induce a higher number of adverse events [31, 74], but the level of evidence remains low.
  Level of evidence 1A (Table 5).
  Median = 8; range = 7–9.
  Expert opinion: appropriate with strong agreement.
24. Intra-articular PRP injections in knee osteoarthritis are a systemically well-tolerated treatment.
  Various systemic adverse reactions have been described after PRP injections, including nausea, tachycardia, headache, syncope, and sweating [51, 89]. These symptoms were transient and resolved within a few days. To date, no serious adverse reactions have been reported in clinical studies or in case reports [89].
  Level of evidence 1A (Table 5).
  Median = 9; rank = 6–9.
  Expert opinion: appropriate with relative agreement.
25. Symptomatic bilateral knee osteoarthritis can be treated at the same time.

**Table 1:**
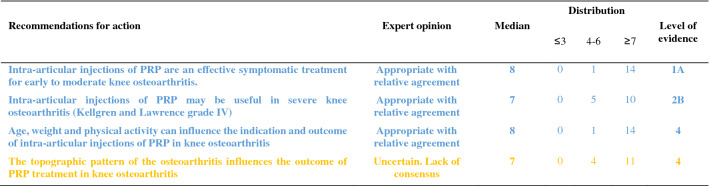
Recommendations related to PRP indications

Color code: blue, recommendation considered appropriate with a relative agreement; orange, recommendation considered uncertain

**Table 2:**
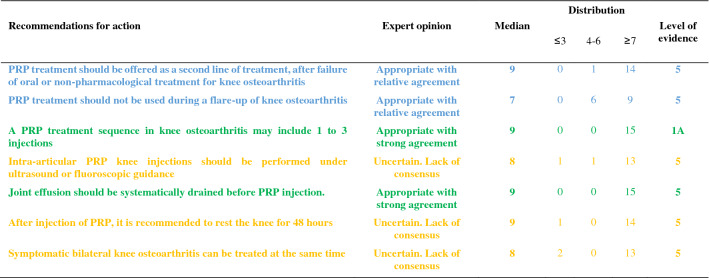
General recommendations

Color code: green, recommendation considered appropriate with a strong agreement; blue, recommendation considered appropriate with a relative agreement; orange, recommendation considered uncertain

**Table 3:**
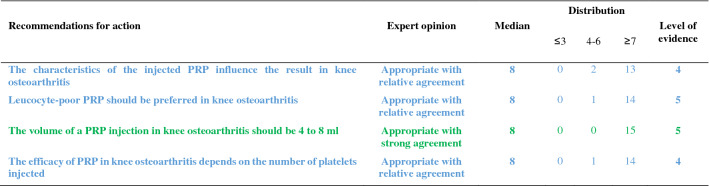
Recommendations related to PRP characteristics

Color code: green, recommendation considered appropriate with a strong agreement; blue, recommendation considered appropriate with a relative agreement

**Table 4:**
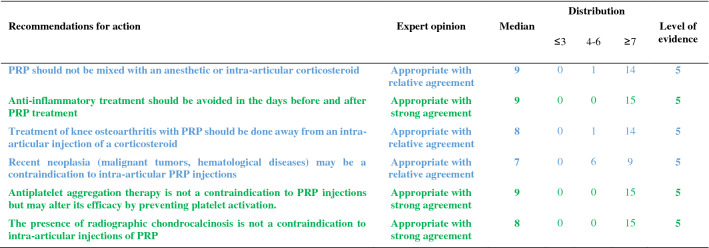
Recommendations related to contraindications and interactions

Color code: green, recommendation considered appropriate with a strong agreement; blue, recommendation considered appropriate with a relative agreement

**Table 5:**
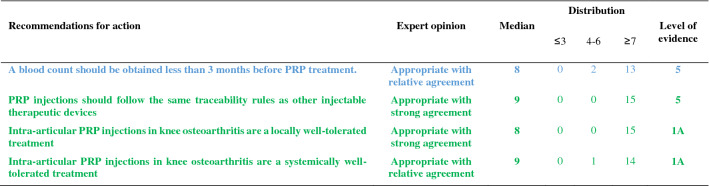
Recommendations related to good practice and adverse events

Color code: green, recommendation considered appropriate with a strong agreement; blue, recommendation considered appropriate with a relative agreement

The simultaneous injection of PRP in both knees was acceptable for the majority of experts, with the exception of 2 experts. The literature does not provide evidence to justify either position. The only risk retained by the experts was a painful episode or even an inflammatory flare-up after injection of PRP, which would be a major functional limitation if both knees were affected.

Level of evidence 5 (Table 2).

Median = 8; rank = 2–9.

Expert opinion: uncertain. Lack of consensus.

## Discussion

PRP in knee OA has experienced a real boom in recent years, which can be seen both in clinical practice and in the scientific literature. The experts have considered that PRP was an effective treatment for symptomatic early or moderate knee OA and might be used in severe forms of knee OA, particularly in cases of contraindications to surgery. They have acknowledged that the level of scientific evidence for end-stage OA was much lower and that PRP have probably a lower symptomatic effect than in earlier stages. Experts agreed that PRP should be only proposed after failure of well-conducted oral and topic symptomatic treatments and appropriate physiotherapy. Nevertheless, PRP is a treatment that remains, to date, costly for the patient and which is in “competition” with other injectable treatments whose symptomatic efficacy is at least partially recognized by many experts. Consequently, the use of PRP rather than injectable CS and HA should be discussed on a case-by-case basis, especially considering that CS injections should be preferred during an acute inflammatory phase.

Even if the number of publications has grown exponentially and now includes several randomized controlled trials and meta-analyses, the significant heterogeneity in the composition of the PRP, the injected volume and the injection protocols makes the evaluation of this treatment difficult and underlines the need for standardization. It was therefore essential for the medical and scientific community to establish practical recommendations, as accurate as possible, on the use of PRP injections for knee OA by experts with clinical and scientific experience in order to standardize clinical practices and mainly avoid misuse. From this work emerged 25 recommendations, the majority of which reached a consensus despite certain methodological limitations and pitfalls which will have to be addressed by further studies. In particular, the experts agreed on the need for larger trials with standard protocols, and simple methodology defined according to the recommendations of international expert groups (OARSI) [67] to increase the level of evidence regarding the use of PRP in knee OA and remove the remaining doubts about the efficacy of PRP. In priority, it is necessary to conduct robust randomized controlled trials comparing intra-articular PRP injections to saline injections. Indeed, the placebo effect, now considered as an integral part of treatment effect (contextual effect) in OA, is a key point to consider when assessing the effect of treatments especially intra-articular injections. The improvement 3 months after intra-articular saline injection was evaluated at 12 points out of 100 on VAS pain and at almost 20 points out of 100 on the total WOMAC score [86] whereas the contextual effect of placebo was estimated at 47% for corticosteroid injections and 82% for hyaluronic acid [100].

Also, authors have considered that certain patient characteristics (age, history, comorbidities, etc.) could influence the composition of PRP and possibly its clinical efficacy. The topography of the cartilage lesions, and specifically the existence of patellofemoral OA, could modulate the effect of PRP, as with other injectable treatments [18, 44]. Nevertheless, some experts pointed out the limitations of the literature on this topic, meaning that the recommendations will have to be updated as new data emerges. Experts stated that the presence of radiographic chondrocalcinosis was not a contraindication, but the potential risk of crystal-induced arthritis following PRP injection justifies that patients be informed before the procedure. Further studies could also help establish a more precise injection protocol (number of injections, frequency, and volume) with a PRP of clearly identified composition. In addition, specific endpoints should be investigated, such as delay before prosthesis surgery or structural effect by direct assessment with X-ray or MRI or indirect assessment with biological biomarkers. Health-economics studies are also warranted in the medium term.

Many unknowns remained about the ideal composition of PRP, but a consensus has emerged for LP-PRP that contains ≤ 1 000 000 platelets per mm^3^ with an injection volume of 4–8 ml. One to three PRP injections should be performed in the same therapeutic cycle, knowing that multiple injections could increase and/or prolong treatment efficacy [37, 42, 48]. There was no available data for a weekly, bi-monthly or monthly frequency. Though it was not considered mandatory, experts have recommended that injections should be guided by ultrasound or fluoroscopy considering that clinical guidance failed in about 10–30%. The question of whether of NSAIDs and platelet aggregation inhibitors should be discontinued regularly arises in practice. Based on scientific data, experts have considered appropriate to temporarily discontinue NSAIDs around the time of PRP injection. Based on empiric evidence, experts have proposed a minimum interruption of one week before and one week after the injection. However, they did not generally recommend discontinuation of platelet aggregation inhibitors due to the potential risk of severe cardiovascular complications. The expert group has considered that PRP injections could therefore be performed under aggregation inhibitors while stressing that their impact on the effectiveness of the treatment was not known to date. As a precaution, experts advised against the use of PRP infiltration in context of solid neoplasia and hemopathy that are neither cured nor in remission or of local knee tumor, although there was no study that determines the neoplastic risks associated with local injection of growth factors. Practitioners should obtain a recent blood count (less than 3 months) before PRP in order to screen for a hematological abnormality that could disrupt the efficacy of PRP (thrombocytopenia) and constitute a contraindication to this treatment. If in doubt, a second opinion from a hematologist could be requested. The experts agreed on the good local and general tolerance of PRP injections, particularly in terms of infection. Patients should be informed of a frequent transient worsening of pain (about 5–10%), which occur rapidly after injection and fade within a few days.

The limitations of this work were inherent in the methodology used since expert opinions made up for the gaps in the literature. Thus, most of the recommendations were effectively based on expert opinions given the lack of available literature data. Also, although the methodology of its study was largely inspired by the recommendations from the French National Authority for Health [39], some of them were not strictly followed, given their heaviness and complexity. Thus, the French National Authority for Health did not head this project and therefore private funding had to be obtained for the organization of the different meetings. In addition, we did not clearly separate the steering committee from the rating group. While a first group of experts was responsible for carrying out the literature review and proposing the recommendations, all 15 experts participated in the votes. Finally, there was no reading group. Nevertheless, we consider that despite these methodological deviations, we have led this consensus by respecting as much as possible the main principles of the methodology proposed by the French National Authority for Health. A potential bias concerned the low heterogeneity of geographic origin of experts. Indeed, all of them originated from French-speaking countries. Although 3 continents were represented with one expert from Morocco and one from Canada, the vast majority of experts were European, mainly from France or neighboring European countries (Belgium, Switzerland). Consequently, most (but not all) experts shared a similar healthcare system and culture model that could influence their opinion and so the recommendations they provided, limiting their international generalization*.* Another limitation of our consensus was the absence of any orthopedic surgeon in the expert group. Indeed, in most French-speaking countries, orthopedic surgeons are mainly involved in late OA stages, especially to discuss surgical management whereas in earlier OA stages, patients are frequently referred by their general practitioners to a rheumatologist or a rehabilitation specialist. However, in many countries, orthopedists are involved at all stages of OA and may have insights into outcomes and alternative treatments not seen by other physician. This could also limit the scope of our recommendations at the international level and constitutes another bias in this study. Finally, authors limited their research to the Medline database, but it was unlikely that any significant studies were missed in their analysis. This work received funding from a private company (RegenLab^®^) for meeting organization (including travel, accommodations and meals). Some experts were also consultant for this company as disclosed at the end of the manuscript. However, the company did not influence the selection of experts, chosen by the head of the GRIP (HB) and the panel of experts remained fully independent through all the process.

## Conclusion

The expert group produced a consensus statement on PRP injections in knee OA, which was considered as an effective treatment for symptomatic early or moderate knee OA after failure of well-conducted oral symptomatic treatment and appropriate physiotherapy and might be used in severe forms of knee OA, particularly in cases of contraindications to surgery. Twenty-five recommendations regarding the indications, the protocol for injections and the possible contraindications and complications were provided. A strong or relative agreement from the experts was obtained for most of the recommendations. However, many of them had a very low level of evidence (Level 5) and were principally based on the clinical experience of the experts. This set of recommendations should harmonize and facilitate the use of IA PRP injections for knee OA.

## Electronic supplementary material

Below is the link to the electronic supplementary material.

Supplementary file1 (PDF 59 kb)

Supplementary file2 (DOCX 14 kb)

## Data Availability

Not applicable.

## References

[1] Abhishek A (2016). Calcium pyrophosphate deposition disease: a review of epidemiologic findings. Curr Opin Rheumatol.

[2] Ahlbäck S, Rydberg J (1980). X-ray classification and examination technics in gonarthrosis. Lakartidningen.

[3] Ahmad HS, Farrag SE, Okasha AE, Kadry AO, Ata TB, Monir AA, Shady I (2018). Clinical outcomes are associated with changes in ultrasonographic structural appearance after platelet-rich plasma treatment for knee osteoarthritis. Int J Rheum Dis.

[4] Assirelli E, Filardo G, Mariani E, Kon E, Roffi A, Vaccaro F, Marcacci M, Facchini A, Pulsatelli L (2015). Effect of two different preparations of platelet-rich plasma on synoviocytes. Knee Surg Sports Traumatol Arthrosc.

[5] Bannuru RR, Osani MC, Vaysbrot EE, Arden NK, Bennell K, Bierma-Zeinstra SMA, Kraus VB, Lohmander LS, Abbott JH, Bhandari M, Blanco FJ, Espinosa R, Haugen IK, Lin J, Mandl LA, Moilanen E, Nakamura N, Snyder-Mackler L, Trojian T, Underwood M, McAlindon TE (2019). OARSI guidelines for the non-surgical management of knee, hip, and polyarticular osteoarthritis. Osteoarthr Cartil.

[6] Berkoff DJ, Miller LE, Block JE (2012). Clinical utility of ultrasound guidance for intra-articular knee injections: a review. Clin Interv Aging.

[7] Braun HJ, Kim HJ, Chu CR, Dragoo JL (2014). The effect of platelet-rich plasma formulations and blood products on human synoviocytes: implications for intra-articular injury and therapy. Am J Sports Med.

[8] Buendía-López D, Medina-Quirós M, Fernández-Villacañas Marín MÁ (2018). Clinical and radiographic comparison of a single LP-PRP injection, a single hyaluronic acid injection and daily NSAID administration with a 52-week follow-up: a randomized controlled trial. J Orthop Traumatol.

[9] Bum Park Y, Ah Choi W, Kim Y-K, Chul Lee S, Hae Lee J (2012). Accuracy of blind versus ultrasound-guided suprapatellar bursal injection. J Clin Ultrasound.

[10] Burns PB, Rohrich RJ, Chung KC (2011). The levels of evidence and their role in evidence-based medicine. Plast Reconstr Surg.

[11] Camurcu Y, Sofu H, Ucpunar H, Kockara N, Cobden A, Duman S (2018). Single-dose intra-articular corticosteroid injection prior to platelet-rich plasma injection resulted in better clinical outcomes in patients with knee osteoarthritis: a pilot study. J Back Musculoskelet Rehabil.

[12] Carofino B, Chowaniec DM, McCarthy MB, Bradley JP, Delaronde S, Beitzel K, Cote MP, Arciero RA, Mazzocca AD (2012). Corticosteroids and local anesthetics decrease positive effects of platelet-rich plasma: an in vitro study on human tendon cells. Arthroscopy.

[13] Cerza F, Carnì S, Carcangiu A, Di Vavo I, Schiavilla V, Pecora A, De Biasi G, Ciuffreda M (2012). Comparison between hyaluronic acid and platelet-rich plasma, intra-articular infiltration in the treatment of gonarthrosis. Am J Sports Med.

[14] Chang K-V, Hung C-Y, Aliwarga F, Wang T-G, Han D-S, Chen W-S (2014). Comparative effectiveness of platelet-rich plasma injections for treating knee joint cartilage degenerative pathology: a systematic review and meta-analysis. Arch Phys Med Rehabil.

[15] Chen CPC, Cheng C-H, Hsu C-C, Lin H-C, Tsai Y-R, Chen J-L (2017). The influence of platelet rich plasma on synovial fluid volumes, protein concentrations, and severity of pain in patients with knee osteoarthritis. Exp Gerontol.

[16] Chouhan DK, Dhillon MS, Patel S, Bansal T, Bhatia A, Kanwat H (2019). Multiple platelet-rich plasma injections versus single platelet-rich plasma injection in early osteoarthritis of the knee: an experimental study in a guinea pig model of early knee osteoarthritis. Am J Sports Med.

[17] Cole BJ, Karas V, Hussey K, Pilz K, Fortier LA (2017). Hyaluronic acid versus platelet-rich plasma: a prospective, double-blind randomized controlled trial comparing clinical outcomes and effects on intra-articular biology for the treatment of knee osteoarthritis. Am J Sports Med.

[18] Conrozier T, Monfort J, Chevalier X, Raman R, Richette P, Diraçoglù D, Bard H, Baron D, Jerosch J, Migliore A, Henrotin Y (2018). EUROVISCO recommendations for optimizing the clinical results of viscosupplementation in osteoarthritis. Cartilage.

[19] Cooper C, Chapurlat R, Al-Daghri N, Herrero-Beaumont G, Bruyère O, Rannou F, Roth R, Uebelhart D, Reginster J-Y (2019). Safety of oral non-selective non-steroidal anti-inflammatory drugs in osteoarthritis: what does the literature say?. Drugs Aging.

[20] Curtis E, Fuggle N, Shaw S, Spooner L, Ntani G, Parsons C, Corp N, Honvo G, Baird J, Maggi S, Dennison E, Bruyère O, Reginster J-Y, Cooper C (2019). Safety of cyclooxygenase-2 inhibitors in osteoarthritis: outcomes of a systematic review and meta-analysis. Drugs Aging.

[21] Dai W-L, Zhou A-G, Zhang H, Zhang J (2017). Efficacy of platelet-rich plasma in the treatment of knee osteoarthritis: a meta-analysis of randomized controlled trials. Arthroscopy.

[22] Dernek B, Kesiktas FN, Duymus TM, Aydin T, Isiksacan N, Diracoglu D, Aksoy C (2017). Effect of platelet concentration on clinical improvement in treatment of early stage-knee osteoarthritis with platelet-rich plasma concentrations. J Phys Ther Sci.

[23] Di Martino A, Di Matteo B, Papio T, Tentoni F, Selleri F, Cenacchi A, Kon E, Filardo G (2018). Platelet-rich plasma versus hyaluronic acid injections for the treatment of knee osteoarthritis: results at 5 years of a double-blind, randomized controlled trial. Am J Sports Med.

[24] Dohan Ehrenfest DM, Rasmusson L, Albrektsson T (2009). Classification of platelet concentrates: from pure platelet-rich plasma (P-PRP) to leucocyte- and platelet-rich fibrin (L-PRF). Trends Biotechnol.

[25] Durant TJS, Dwyer CR, McCarthy MBR, Cote MP, Bradley JP, Mazzocca AD (2017). Protective nature of platelet-rich plasma against chondrocyte death when combined with corticosteroids or local anesthetics. Am J Sports Med.

[26] Duymus TM, Mutlu S, Dernek B, Komur B, Aydogmus S, Kesiktas FN (2017). Choice of intra-articular injection in treatment of knee osteoarthritis: platelet-rich plasma, hyaluronic acid or ozone options. Knee Surg Sports Traumatol Arthrosc.

[27] Evanson JR, Guyton MK, Oliver DL, Hire JM, Topolski RL, Zumbrun SD, McPherson JC, Bojescul JA (2014). Gender and age differences in growth factor concentrations from platelet-rich plasma in adults. Mil Med.

[28] Eymard F, Chevalier X, Conrozier T (2017). Obesity and radiological severity are associated with viscosupplementation failure in patients with knee osteoarthritis. J Orthop Res.

[29] Fawzy RM, Hashaad NI, Mansour AI (2017). Decrease of serum biomarker of type II collagen degradation (Coll2-1) by intra-articular injection of an autologous plasma-rich-platelet in patients with unilateral primary knee osteoarthritis. Eur J Rheumatol.

[30] Fice MP, Miller JC, Christian R, Hannon CP, Smyth N, Murawski CD, Cole BJ, Kennedy JG (2019). The role of platelet-rich plasma in cartilage pathology: an updated systematic review of the basic science evidence. Arthroscopy.

[31] Filardo G, Kon E, Pereira Ruiz MT, Vaccaro F, Guitaldi R, Di Martino A, Cenacchi A, Fornasari PM, Marcacci M (2012). Platelet-rich plasma intra-articular injections for cartilage degeneration and osteoarthritis: single- versus double-spinning approach. Knee Surg Sports Traumatol Arthrosc.

[32] Filardo G, Kon E, DI Matteo B, DI Marino A, Sessa A, Merli ML, Marcacci M (2013). Leukocyte-poor PRP application for the treatment of knee osteoarthritis. Joints.

[33] Filardo G, Di Matteo B, Di Martino A, Merli ML, Cenacchi A, Fornasari P, Marcacci M, Kon E (2015). Platelet-rich plasma intra-articular knee injections show no superiority versus viscosupplementation: a randomized controlled trial. Am J Sports Med.

[34] Fitzpatrick J, Bulsara MK, McCrory PR, Richardson MD, Zheng MH (2017). Analysis of platelet-rich plasma extraction: variations in platelet and blood components between 4 common commercial kits. Orthop J Sports Med.

[35] Gato-Calvo L, Magalhaes J, Ruiz-Romero C, Blanco FJ, Burguera EF (2019). Platelet-rich plasma in osteoarthritis treatment: review of current evidence. Ther Adv Chronic Dis.

[36] Glass F, Lippton H, Kadowitz PJ (1980). Differential effects of local anesthetics and propranolol on arachidonic acid and adenosine 5’-diphosphate-induced aggregation in rabbit platelets. Prostaglandins Med.

[37] Görmeli G, Görmeli CA, Ataoglu B, Çolak C, Aslantürk O, Ertem K (2017). Multiple PRP injections are more effective than single injections and hyaluronic acid in knees with early osteoarthritis: a randomized, double-blind, placebo-controlled trial. Knee Surg Sports Traumatol Arthrosc.

[38] Grant GJ, Ramanathan S, Patel N, Turndorf H (1989). The effects of local anesthetics on maternal and neonatal platelet function. Acta Anaesthesiol Scand.

[39] Guide méthodologique consensus formalise.pdf [Internet]. Available from: https://www.has-sante.fr/upload/docs/application/pdf/2011-01/guide_methodologique_consensus_formalise.pdf

[40] Guillibert C, Charpin C, Raffray M, Benmenni A, Dehaut F-X, El Ghobeira G, Giorgi R, Magalon J, Arniaud D (2019). Single injection of high volume of autologous pure PRP provides a significant improvement in knee osteoarthritis: a prospective routine care study. Int J Mol Sci.

[41] Han Y, Huang H, Pan J, Lin J, Zeng L, Liang G, Yang W, Liu J (2019). Meta-analysis comparing platelet-rich plasma vs hyaluronic acid injection in patients with knee osteoarthritis. Pain Med.

[42] Huang P-H, Wang C-J, Chou W-Y, Wang J-W, Ko J-Y (2017). Short-term clinical results of intra-articular PRP injections for early osteoarthritis of the knee. Int J Surg.

[43] Huang Y, Liu X, Xu X, Liu J (2019). Intra-articular injections of platelet-rich plasma, hyaluronic acid or corticosteroids for knee osteoarthritis: a prospective randomized controlled study. Orthopade.

[44] Jang S-J, Kim J-D, Cha S-S (2013). Platelet-rich plasma (PRP) injections as an effective treatment for early osteoarthritis. Eur J Orthop Surg Traumatol.

[45] Jo CH, Lee SY, Yoon KS, Shin S (2017). Effects of platelet-rich plasma with concomitant use of a corticosteroid on tenocytes from degenerative rotator cuff tears in interleukin 1β-induced tendinopathic conditions. Am J Sports Med.

[46] Joshi Jubert N, Rodríguez L, Reverté-Vinaixa MM, Navarro A (2017). Platelet-rich plasma injections for advanced knee osteoarthritis: a prospective, randomized, double-blinded clinical trial. Orthop J Sports Med.

[47] Kanchanatawan W, Arirachakaran A, Chaijenkij K, Prasathaporn N, Boonard M, Piyapittayanun P, Kongtharvonskul J (2016). Short-term outcomes of platelet-rich plasma injection for treatment of osteoarthritis of the knee. Knee Surg Sports Traumatol Arthrosc.

[48] Kavadar G, Demircioglu DT, Celik MY, Emre TY (2015). Effectiveness of platelet-rich plasma in the treatment of moderate knee osteoarthritis: a randomized prospective study. J Phys Ther Sci.

[49] Kellgren JH, Lawrence JS (1957). Radiological assessment of osteo-arthrosis. Ann Rheum Dis.

[50] Khatab S, van Buul GM, Kops N, Bastiaansen-Jenniskens YM, Bos PK, Verhaar JA, van Osch GJ (2018). Intra-articular injections of platelet-rich plasma releasate reduce pain and synovial inflammation in a mouse model of osteoarthritis. Am J Sports Med.

[51] Khoshbin A, Leroux T, Wasserstein D, Marks P, Theodoropoulos J, Ogilvie-Harris D, Gandhi R, Takhar K, Lum G, Chahal J (2013). The efficacy of platelet-rich plasma in the treatment of symptomatic knee osteoarthritis: a systematic review with quantitative synthesis. Arthroscopy.

[52] Kolasinski SL, Neogi T, Hochberg MC, Oatis C, Guyatt G, Block J, Callahan L, Copenhaver C, Dodge C, Felson D, Gellar K, Harvey WF, Hawker G, Herzig E, Kwoh CK, Nelson AE, Samuels J, Scanzello C, White D, Wise B, Altman RD, DiRenzo D, Fontanarosa J, Giradi G, Ishimori M, Misra D, Shah AA, Shmagel AK, Thoma LM, Turgunbaev M, Turner AS, Reston J (2020). 2019 American college of rheumatology/arthritis foundation guideline for the management of osteoarthritis of the hand, hip, and knee. Arthritis Rheumatol.

[53] Kon E, Buda R, Filardo G, Di Martino A, Timoncini A, Cenacchi A, Fornasari PM, Giannini S, Marcacci M (2010). Platelet-rich plasma: intra-articular knee injections produced favorable results on degenerative cartilage lesions. Knee Surg Sports Traumatol Arthrosc.

[54] Kon E, Mandelbaum B, Buda R, Filardo G, Delcogliano M, Timoncini A, Fornasari PM, Giannini S, Marcacci M (2011). Platelet-rich plasma intra-articular injection versus hyaluronic acid viscosupplementation as treatments for cartilage pathology: from early degeneration to osteoarthritis. Arthroscopy.

[55] Laudy ABM, Bakker EWP, Rekers M, Moen MH (2015). Efficacy of platelet-rich plasma injections in osteoarthritis of the knee: a systematic review and meta-analysis. Br J Sports Med.

[56] Lin K-Y, Yang C-C, Hsu C-J, Yeh M-L, Renn J-H (2019). Intra-articular injection of platelet-rich plasma is superior to hyaluronic acid or saline solution in the treatment of mild to moderate knee osteoarthritis: a randomized, double-blind, triple-parallel, placebo-controlled clinical trial. Arthroscopy.

[57] Liu X, Wang L, Ma C, Wang G, Zhang Y, Sun S (2019). Exosomes derived from platelet-rich plasma present a novel potential in alleviating knee osteoarthritis by promoting proliferation and inhibiting apoptosis of chondrocyte via Wnt/β-catenin signaling pathway. J Orthop Surg.

[58] Louis ML, Magalon J, Jouve E, Bornet CE, Mattei JC, Chagnaud C, Rochwerger A, Veran J, Sabatier F (2018). Growth factors levels determine efficacy of platelets rich plasma injection in knee osteoarthritis: a randomized double blind noninferiority trial compared with viscosupplementation. Arthroscopy.

[59] Ludwig HC, Birdwhistell KE, Brainard BM, Franklin SP (2017). Use of a cyclooxygenase-2 inhibitor does not inhibit platelet activation or growth factor release from platelet-rich plasma. Am J Sports Med.

[60] Magalon J, Bausset O, Serratrice N, Giraudo L, Aboudou H, Veran J, Magalon G, Dignat-Georges F, Sabatier F (2014). Characterization and comparison of 5 platelet-rich plasma preparations in a single-donor model. Arthroscopy.

[61] Magalon J, Velier M, Francois P, Graiet H, Veran J, Sabatier F (2017). Comment on “responders to platelet-rich plasma in osteoarthritis: a technical analysis”. BioMed Res Int.

[62] Mannava S, Whitney KE, Kennedy MI, King J, Dornan GJ, Klett K, Chahla J, Evans TA, Huard J, LaPrade RF (2019). The influence of naproxen on biological factors in leukocyte-rich platelet-rich plasma: a prospective comparative study. Arthroscopy.

[63] Mariani E, Canella V, Cattini L, Kon E, Marcacci M, Di Matteo B, Pulsatelli L, Filardo G (2016). Leukocyte-rich platelet-rich plasma injections do not up-modulate intra-articular pro-inflammatory cytokines in the osteoarthritic knee. PLoS ONE.

[64] Mariani E, Roffi A, Cattini L, Pulsatelli L, Assirelli E, Krishnakumar GS, Cenacchi A, Kon E, Filardo G (2020). Release kinetic of pro- and anti-inflammatory biomolecules from platelet-rich plasma and functional study on osteoarthritis synovial fibroblasts. Cytotherapy.

[65] Marty M, Hilliquin P, Rozenberg S, Valat JP, Vignon E, Coste P, Savarieau B, Allaert FA (2009). Validation of the KOFUS (knee osteoarthritis flare-ups score). Jt Bone Spine.

[66] Mazzocca AD, McCarthy MBR, Chowaniec DM, Cote MP, Romeo AA, Bradley JP, Arciero RA, Beitzel K (2012). Platelet-rich plasma differs according to preparation method and human variability. J Bone Jt Surg Am.

[67] McAlindon TE, Driban JB, Henrotin Y, Hunter DJ, Jiang G-L, Skou ST, Wang S, Schnitzer T (2015). OARSI clinical trials recommendations: design, conduct, and reporting of clinical trials for knee osteoarthritis. Osteoarthr Cartil.

[68] Meheux CJ, McCulloch PC, Lintner DM, Varner KE, Harris JD (2016). Efficacy of intra-articular platelet-rich plasma injections in knee osteoarthritis: a systematic review. Arthroscopy.

[69] Milants C, Bruyère O, Kaux J-F (2017). Responders to platelet-rich plasma in osteoarthritis: a technical analysis. BioMed Res Int.

[70] Montañez-Heredia E, Irízar S, Huertas PJ, Otero E, Del Valle M, Prat I, Díaz-Gallardo MS, Perán M, Marchal JA, Hernandez-Lamas MDC (2016). Intra-articular injections of platelet-rich plasma versus hyaluronic acid in the treatment of osteoarthritic knee pain: a randomized clinical trial in the context of the spanish national health care system. Int J Mol Sci.

[71] Moussa M, Lajeunesse D, Hilal G, El Atat O, Haykal H, Serhal R, Chalhoub A, Khalil C, Alaaeddine N (2017). Platelet rich plasma (PRP) induces chondroprotection via increasing autophagy, anti-inflammatory markers, and decreasing apoptosis in human osteoarthritic cartilage. Exp Cell Res.

[72] O’Donnell C, Migliore E, Grandi FC, Koltsov J, Lingampalli N, Cisar C, Indelli PF, Sebastiano V, Robinson WH, Bhutani N, Chu CR (2019). Platelet-rich plasma (PRP) from older males with knee osteoarthritis depresses chondrocyte metabolism and upregulates inflammation. J Orthop Res.

[73] Ornetti P, Nourissat G, Berenbaum F, Sellam J, Richette P, Chevalier X, under the aegis of the Osteoarthritis Section of the French Society for Rheumatology (Société Française de Rhumatologie, SFR) (2016). Does platelet-rich plasma have a role in the treatment of osteoarthritis?. Jt Bone Spine.

[74] Patel S, Dhillon MS, Aggarwal S, Marwaha N, Jain A (2013). Treatment with platelet-rich plasma is more effective than placebo for knee osteoarthritis: a prospective, double-blind, randomized trial. Am J Sports Med.

[75] Pinto LMA, Pereira R, de Paula E, de Nucci G, Santana MHA, Donato JL (2004). Influence of liposomal local anesthetics on platelet aggregation in vitro. J Liposome Res.

[76] Qiao J, An N, Ouyang X (2017). Quantification of growth factors in different platelet concentrates. Platelets.

[77] Raeissadat SA, Rayegani SM, Hassanabadi H, Fathi M, Ghorbani E, Babaee M, Azma K (2015). Knee osteoarthritis injection choices: platelet- rich plasma (PRP) versus hyaluronic acid (a one-year randomized clinical trial). Clin Med Insights Arthritis Musculoskelet Disord.

[78] Raeissadat SA, Rayegani SM, Ahangar AG, Abadi PH, Mojgani P, Ahangar OG (2017). Efficacy of intra-articular injection of a newly developed plasma rich in growth factor (PRGF) versus hyaluronic acid on pain and function of patients with knee osteoarthritis: a single-blinded randomized clinical trial. Clin Med Insights Arthritis Musculoskelet Disord.

[79] Raeissadat SA, Ghorbani E, Sanei Taheri M, Soleimani R, Rayegani SM, Babaee M, Payami S (2020). MRI changes after platelet rich plasma injection in knee osteoarthritis (randomized clinical trial). J Pain Res.

[80] Rastogi AK, Davis KW, Ross A, Rosas HG (2016). Fundamentals of joint injection. AJR Am J Roentgenol.

[81] Riboh JC, Saltzman BM, Yanke AB, Fortier L, Cole BJ (2016). Effect of leukocyte concentration on the efficacy of platelet-rich plasma in the treatment of knee osteoarthritis. Am J Sports Med.

[82] Richette P, Bardin T, Doherty M (2009). An update on the epidemiology of calcium pyrophosphate dihydrate crystal deposition disease. Rheumatology.

[83] Richette P, Latourte A, Frazier A (2015). Safety and efficacy of paracetamol and NSAIDs in osteoarthritis: which drug to recommend?. Expert Opin Drug Saf.

[84] Saccomanno MF, Donati F, Careri S, Bartoli M, Severini G, Milano G (2016). Efficacy of intra-articular hyaluronic acid injections and exercise-based rehabilitation programme, administered as isolated or integrated therapeutic regimens for the treatment of knee osteoarthritis. Knee Surg Sports Traumatol Arthrosc.

[85] Sadabad HN, Behzadifar M, Arasteh F, Behzadifar M, Dehghan HR (2016). Efficacy of platelet-rich plasma versus hyaluronic acid for treatment of knee osteoarthritis: a systematic review and meta-analysis. Electron Phys.

[86] Saltzman BM, Leroux T, Meyer MA, Basques BA, Chahal J, Bach BR, Yanke AB, Cole BJ (2017). The therapeutic effect of intra-articular normal saline injections for knee osteoarthritis: a meta-analysis of evidence level 1 studies. Am J Sports Med.

[87] Sanchez C, Pesesse L, Gabay O, Delcour J-P, Msika P, Baudouin C, Henrotin YE (2012). Regulation of subchondral bone osteoblast metabolism by cyclic compression. Arthritis Rheum.

[88] Sánchez M, Fiz N, Azofra J, Usabiaga J, Aduriz Recalde E, Garcia Gutierrez A, Albillos J, Gárate R, Aguirre JJ, Padilla S, Orive G, Anitua E (2012). A randomized clinical trial evaluating plasma rich in growth factors (PRGF-Endoret) versus hyaluronic acid in the short-term treatment of symptomatic knee osteoarthritis. Arthroscopy.

[89] Shen L, Yuan T, Chen S, Xie X, Zhang C (2017). The temporal effect of platelet-rich plasma on pain and physical function in the treatment of knee osteoarthritis: systematic review and meta-analysis of randomized controlled trials. J Orthop Surg.

[90] Smith PA (2016). Intra-articular autologous conditioned plasma injections provide safe and efficacious treatment for knee osteoarthritis: an FDA-sanctioned, randomized, double-blind, placebo-controlled clinical trial. Am J Sports Med.

[91] Su K, Bai Y, Wang J, Zhang H, Liu H, Ma S (2018). Comparison of hyaluronic acid and PRP intra-articular injection with combined intra-articular and intraosseous PRP injections to treat patients with knee osteoarthritis. Clin Rheumatol.

[92] Sundman EA, Cole BJ, Karas V, Della Valle C, Tetreault MW, Mohammed HO, Fortier LA (2014). The anti-inflammatory and matrix restorative mechanisms of platelet-rich plasma in osteoarthritis. Am J Sports Med.

[93] Taniguchi Y, Yoshioka T, Sugaya H, Gosho M, Aoto K, Kanamori A, Yamazaki M (2019). Growth factor levels in leukocyte-poor platelet-rich plasma and correlations with donor age, gender, and platelets in the Japanese population. J Exp Orthop.

[94] Vaquerizo V, Plasencia MÁ, Arribas I, Seijas R, Padilla S, Orive G, Anitua E (2013). Comparison of intra-articular injections of plasma rich in growth factors (PRGF-Endoret) versus Durolane hyaluronic acid in the treatment of patients with symptomatic osteoarthritis: a randomized controlled trial. Arthroscopy.

[95] Vilchez-Cavazos F, Millán-Alanís JM, Blázquez-Saldaña J, Álvarez-Villalobos N, Peña-Martínez VM, Acosta-Olivo CA, Simental-Mendía M (2019). Comparison of the clinical effectiveness of single versus multiple injections of platelet-rich plasma in the treatment of knee osteoarthritis: a systematic review and meta-analysis. Orthop J Sports Med.

[96] Watson SP, Bahou WF, Fitzgerald D, Ouwehand W, Rao AK, Leavitt AD (2005). Mapping the platelet proteome: a report of the ISTH platelet physiology subcommittee. J Thromb Haemost.

[97] Xiong G, Lingampalli N, Koltsov JCB, Leung LL, Bhutani N, Robinson WH, Chu CR (2018). Men and women differ in the biochemical composition of platelet-rich plasma. Am J Sports Med.

[98] Xu Z, Luo J, Huang X, Wang B, Zhang J, Zhou A (2017). Efficacy of platelet-rich plasma in pain and self-report function in knee osteoarthritis: a best-evidence synthesis. Am J Phys Med Rehabil.

[99] Yin W-J, Xu H-T, Sheng J-G, An Z-Q, Guo S-C, Xie X-T, Zhang C-Q (2016). Advantages of pure platelet-rich plasma compared with leukocyte- and platelet-rich plasma in treating rabbit knee osteoarthritis. Med Sci Monit.

[100] Zhang W (2019). The powerful placebo effect in osteoarthritis. Clin Exp Rheumatol.

[101] Zhang H, Bai Y, Liu C, Jin S, Su K, Liu Y, Lü Z (2017). Effect of intra-articular injection of platelet-rich plasma on interleukin-17 expression in synovial fluid and venous plasma of knee osteoarthritis patients. Zhongguo Xiu Fu Chong Jian Wai Ke Za Zhi.

[102] Zhang H-F, Wang C-G, Li H, Huang Y-T, Li Z-J (2018). Intra-articular platelet-rich plasma versus hyaluronic acid in the treatment of knee osteoarthritis: a meta-analysis. Drug Des Devel Ther.

