# Correction to: Intra‑articular injections of platelet‑rich plasma in symptomatic knee osteoarthritis: a consensus statement from French‑speaking experts

**DOI:** 10.1007/s00167-020-06331-8

**Published:** 2020-10-24

**Authors:** Florent Eymard, Paul Ornetti, Jérémy Maillet, Éric Noel, Philippe Adam, Virginie Legré-Boyer, Thierry Boyer, Fadoua Allali, Vincent Gremeaux, Jean-François Kaux, Karine Louati, Martin Lamontagne, Fabrice Michel, Pascal Richette, Hervé Bard, the GRIP (Groupe de Recherche sur les Injections de PRP, PRP Injection Research Group)

**Affiliations:** 1 Department of Rheumatology, AP-HP Henri Mondor Hospital, 94010 Créteil Cedex, France; 2 Department of Rheumatology, Plateforme d’Investigations Technologiques Dijon University Hospital, INSERM 1093 CAPS, Dijon, France; 3 Department of Rheumatology, AP-HP Lariboisière Hospital, 75010 Paris, France; 4 Santy Orthopedic Center, 69008 Lyon, France; 5 Imaging Department, Medipole Garonne Sport Clinic, 31100 Toulouse, France; 6 American Hospital Paris, 92200 Neuilly-sur-Seine, France; 7 IAL Nollet, 75017 Paris, France; 8 Department of Rheumatology, El Ayachi Hospital, Salé, Morocco; 9 Sport Medicine Unit, Division of Physical Medicine and Rehabilitation, Swiss Olympic Medical Center, Lausanne University Hospital, Lausanne, Switzerland; 10 Physical, Rehabilitation Medicine and Sports Traumatology, SportS2, FIFA Medical Centre of Excellence, IOC Research for Prevention of Injury and Protection of Athlete Health, FIMS Clinical Centre of Sports Medicine, University and University Hospital of Liège, 4000 Liège, Belgium; 11 Department of Rheumatology, AP-HP Saint-Antoine Hospital, 75012 Paris, France; 12 Montreal University Hospital Center, Montreal, Canada; 13 Physical Medicine and Rehabilitation Department, CHRU hôpital Jean-Minjoz, 25000 Besançon, France; 14 Department of Rheumatology, AP-HP Lariboisière Hospital, 75010 Paris, France; 15 Cabinet médical Vaudoyer, 75007 Paris, France

## Correction to: Knee Surgery, Sports Traumatology, Arthroscopy 10.1007/s00167-020-06102-5

The article Intra‑articular injections of platelet‑rich plasma in symptomatic knee osteoarthritis: a consensus statement from French‑speaking experts, written by Florent Eymard, Paul Ornetti, Jérémy Maillet, Éric Noel, Philippe Adam, Virginie Legre-Boyer, Thierry Boyer, Fadoua Allali, Vincent Gremeaux, Jean-Francois Kaux, Karine Louati, Martin Lamontagne, Fabrice Michel, Pascal Richette, Hervé Bard on behalf of the GRIP (Groupe de Recherche sur les Injections de PRP, PRP Injection Research Group), was originally published electronically on the publisher’s internet portal on 24 June 2020 without open access. With the author(s)’ decision to opt for Open Choice the copyright of the article changed on 25 October 2020 to © The Author(s) 2020 and the article is forthwith distributed under a Creative Commons Attribution 4.0 International License, which permits use, sharing, adaptation, distribution and reproduction in any medium or format, as long as you give appropriate credit to the original author(s) and the source, provide a link to the Creative Commons licence, and indicate if changes were made. The images or other third party material in this article are included in the article’s Creative Commons licence, unless indicated otherwise in a credit line to the material. If material is not included in the article’s Creative Commons licence and your intended use is not permitted by statutory regulation or exceeds the permitted use, you will need to obtain permission directly from the copyright holder. To view a copy of this licence, visit https://creativecommons.org/licenses/by/4.0.

The original article has been corrected.

