# Supplementary material for: Intra-articular injections of platelet-rich plasma in symptomatic knee osteoarthritis: a consensus statement from French-speaking experts
Source: Knee Surg Sports Traumatol Arthrosc. 2020 Jun 24;29(10):3195–210. doi: 10.1007/s00167-020-06102-5 (PMC8458198; doi:10.1007/s00167-020-06102-5)
Supplement: Supplementary file 1 — Supplementary file1 (PDF 59 kb) [file 167_2020_6102_MOESM1_ESM.pdf]

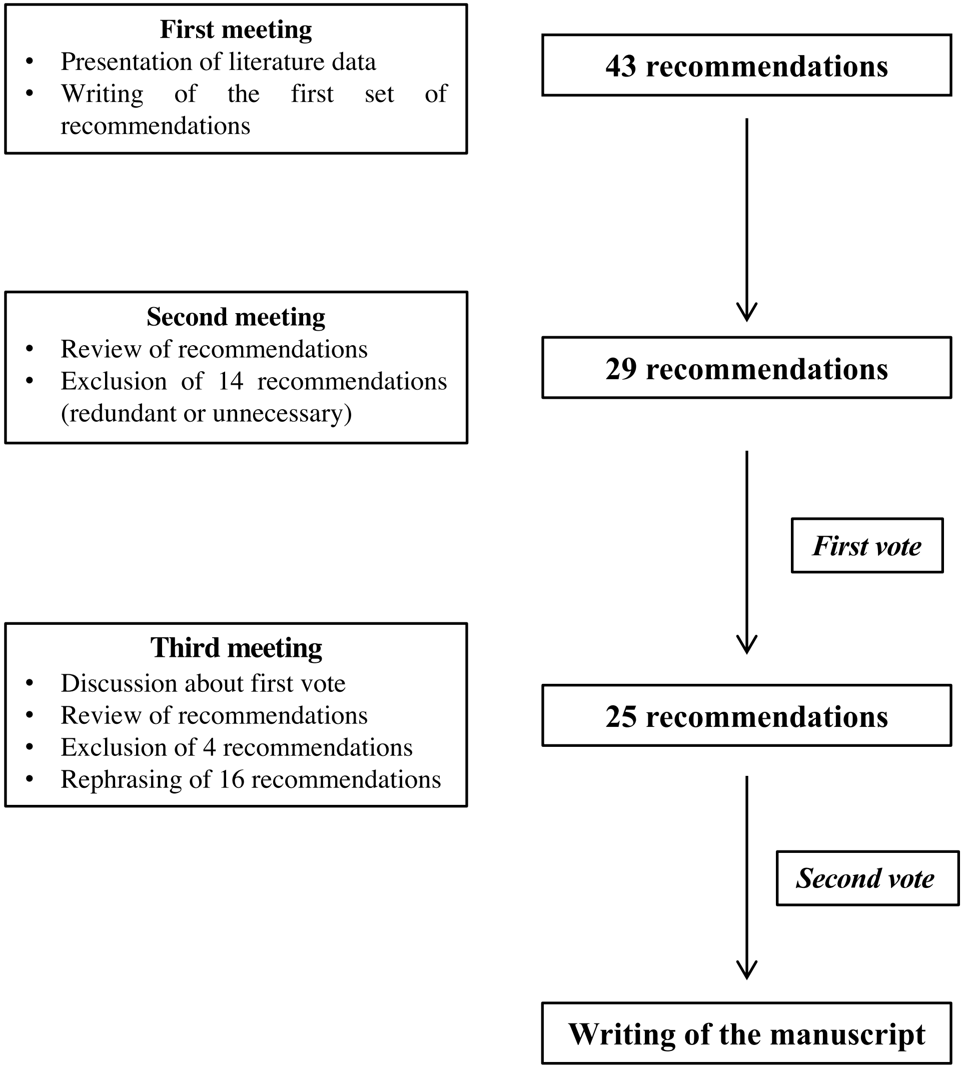
First meeting
Presentation of literature data
Writing of the first set of recommendations
43 recommendations
Second meeting
Review of recommendations
Exclusion of 14 recommendations (redundant or unnecessary)
29 recommendations
First vote
Third meeting
Discussion about first vote
Review of recommendations
Exclusion of 4 recommendations
Rephrasing of 16 recommendations
25 recommendations
Second vote
Writing of the manuscript
