# Supplementary material for: Intra-articular injections of platelet-rich plasma in symptomatic knee osteoarthritis: a consensus statement from French-speaking experts
Source: Knee Surg Sports Traumatol Arthrosc. 2020 Jun 24;29(10):3195–210. doi: 10.1007/s00167-020-06102-5 (PMC8458198; doi:10.1007/s00167-020-06102-5)
Supplement: Supplementary file 2 — Supplementary file2 (DOCX 14 kb) [file 167_2020_6102_MOESM2_ESM.docx]

**Supplementary Table 1. Recommendations excluded before the first vote**

| **Recommendations** | **Level of evidence** |
| --- | --- |
| **The time interval between each injection should be approximately one month** | **5** |
| **A count of the red blood cells, leukocytes and platelets contained in the injected PRP should be systematic** | **5** |
| **PRP injections into the knee do not require systematic guidance** | **5** |
| **Primary or secondary osteochondromatosis is a contraindication** | **5** |
| **An anti-coagulant treatment constitutes a contraindication to PRP injections** | **5** |
| **MGUS and recent hemopathy are contraindications to PRP injections** | **5** |
| **After intra-articular injection of PRP, mobilization of the joint is desirable** | **5** |
| **Any placebo-controlled study concerning PRP injections should include a blinded control group with a blood sample and intra-articular injection** | **1A** |
| **Better results are obtained by association with hyaluronic acid** | **1B** |
| **A hematologist should be integrated into the reference centers for PRP treatments** | **5** |
| **PRP injections in knee OA are an alternative to hyaluronic acid injections** | **1A** |
| **PRP injections are a positive indication for osteoarthritis and not a default treatment** | **1A** |
| **The presence of varus or valgus decompensation while walking influences the result** | **5** |
| **The injection protocol is validated by the evidence-based medicine** | **5** |

**Supplementary Table 2. Recommendations excluded before the second vote**

| **Recommendations** | **Level of evidence** |
| --- | --- |
| **Heterogeneity of PRP prevent extrapolation of the results for one specific PRP to all other PRP** | **5** |
| **The use of a tourniquet for sampling should be prohibited** | **5** |
| **NaCl is the best placebo for controlled studies (Level of evidence 1A)** | **1A** |
| **PRP injections could have a different placebo effect as compared to other injectable products in knee OA due to their autologous origin (Level of evidence 5)** | **5** |
